# Rest for the Weary

**Published:** 1870-04

**Authors:** 


### REST FOR THE WEARY.

One of the greatest physiological crimes of our country is that its people do not get rest enough, do not sleep enough. I saw a man yesterday, or rather the skeleton of a man, for he was at death's door, yet in the prime of life; most of his time was spent in bed. Said he, "When I was a young man I was full of life; I engaged in pleasure and in fun of every form. I delighted in parties; was often invited out several times a week, almost every night. Sometimes at these I would remain long past midnight, and after two or three hours' sleep would go to my business as if nothing had ever happened, then when almost worn out I would take my yacht as a recreation; this was very exciting, and it seemed to me that I couldn't sleep, did not want to sleep. At last my constitution broke down. I travelled over half the world, went to sea for several years, was always temperate, had no bad habits, and here I am."

Every one, of even slight observation, must have noticed that if the usual sleep at night has been disturbed or curtailed, there is a general lassitude of body and mind the next day; there is a feeling of tiredness of body and want of mental elasticity. This condition of things keeps persons liable to take cold from slight causes, to imbibe disease from every source, and, before aware of it, some settled malady has fixed itself in the system, which is but the beginning of the end.

All out-door laborers, all persons who study hard, should get all the sleep the body can possibly take, for sleep is the perfection of rest. A

man who gets seven or eight hours of good, sound, refreshing sleep will do three times the work of one who sleeps but four hours, whether the work be of body or brain. Our wives are old at thirty, mainly because they are deprived of necessary sleep in connection with the nursing and care of children, and the premature oldness or debility is reflected to the children born to her, and they grow up sickly, puny, and diseased.

It is nothing short of a cruelty and a murder to allow school children to sit up late at night, or to hurry them from their beds in the morning. The foundation of inflammation of the brain, of water on the brain, of various forms of convulsions and fits and other alarming maladies, are very often laid in the over-stimulus of the brain in preparing for school examinations, and in performing feats of memory in committing to memory hymns, Scripture verses, and speeches for purposes of declamation.

The rule for every worker, for every student, is imperative; is of universal application and appropriateness. Get all the sleep your body will take, go to bed at a regular hour, and never allow being waked up in the morning as a habit. When the system has got sleep enough to repair the draughts made on it by previous labor, it will wake itself up, just as when in eating we have taken enough food to repair the wants of the system, and to lay in a stock for the next few hours' use, the further appetite for food ceases.

It will be no loss of time in the long run, especially to those who have passed the fiftieth year, to remain in bed of mornings, after waking up, until the feeling of tiredness has passed from the limbs. All know what an effort it is to get out of bed the moment we wake. Notice how hard it is to get our children to leave their beds, even after we have waked them up ; it is because a wise instinct dictates another kind of rest, different from sleep, a kind of waking sleep, as imperatively necessary as the other. But parents should see to it, that the children are put to bed early enough to give them full time for both of these sleeps ; one a rest for the brain, the other a rest for the body.

Let the observant reader put these things to a practical test for himself, and let the result shape his future habits, by getting up one morning the moment he wakes up, and on the next remain in bed without going to sleep a second time, until there is a desire to get up, until the feeling of tiredness has passed from the limbs.

